# Neural Mechanisms Underlying Motivation of Mental Versus Physical Effort

**DOI:** 10.1371/journal.pbio.1001266

**Published:** 2012-02-21

**Authors:** Liane Schmidt, Maël Lebreton, Marie-Laure Cléry-Melin, Jean Daunizeau, Mathias Pessiglione

**Affiliations:** 1 Motivation, Brain and Behavior (MBB) Team, Institut du Cerveau et de la Moelle Epinière (ICM), Paris, France; 2 INSERM UMRS 975, CNRS UMR 7225, Université Pierre et Marie Curie (UPMC–Paris 6), Paris, France; 3 Centre de NeuroImagerie de Recherche (CENIR), Hôpital Pitié-Salpêtrière, Paris, France; 4 Laboratory for Social and Neural Systems Research, University of Zurich, Switzerland; California Institute of Technology, United States of America

## Abstract

Mental and physical efforts, such as paying attention and lifting weights, have been shown to involve different brain systems. These cognitive and motor systems, respectively, include cortical networks (prefronto-parietal and precentral regions) as well as subregions of the dorsal basal ganglia (caudate and putamen). Both systems appeared sensitive to incentive motivation: their activity increases when we work for higher rewards. Another brain system, including the ventral prefrontal cortex and the ventral basal ganglia, has been implicated in encoding expected rewards. How this motivational system drives the cognitive and motor systems remains poorly understood. More specifically, it is unclear whether cognitive and motor systems can be driven by a common motivational center or if they are driven by distinct, dedicated motivational modules. To address this issue, we used functional MRI to scan healthy participants while performing a task in which incentive motivation, cognitive, and motor demands were varied independently. We reasoned that a common motivational node should (1) represent the reward expected from effort exertion, (2) correlate with the performance attained, and (3) switch effective connectivity between cognitive and motor regions depending on task demand. The ventral striatum fulfilled all three criteria and therefore qualified as a common motivational node capable of driving both cognitive and motor regions of the dorsal striatum. Thus, we suggest that the interaction between a common motivational system and the different task-specific systems underpinning behavioral performance might occur within the basal ganglia.

## Author Summary

Incentive motivation refers to the process in the brain by which we translate the expectation of a potential reward into the effort required to do an action, as for instance when the expected paycheck brings the employee to work. Different types of effort can be implemented in everyday life, some being more cognitive, like paying attention, and others more motor-involved, like lifting weights. Reward, cognitive, and motor representations are known to rely on distinct regions of the frontal cortex and basal ganglia. However, how expected rewards motivate these different types of efforts remains poorly understood. Here, we addressed this question by developing a functional neuroimaging approach where we independently varied a monetary reward as well as the cognitive and motor demand of the task. Our results suggest that the expectation of a reward is encoded in the ventral striatum, which can then drive either the motor or cognitive part of the dorsal striatum, depending on the task, in order to boost behavioral performance. We conclude that intra-striatal effective connectivity may explain how both motor and cognitive efforts can be driven by a single motivational module.

## Introduction

There are many situations in life where the outcome depends on how much effort we exert. For instance, an athlete who wishes to win a marathon must train hard. The athlete is likely to train even harder if the race is associated with higher outcomes in terms of social prestige or monetary prize. Incentive motivation refers to the set of processes that translate higher expected rewards into higher effort exertion [1]. These processes include forming a subjective representation of potential reward magnitude capable of boosting behavioral performance. In the previous example, the incentives would boost physical effort, but we can imagine situations where mental effort, rather than physical effort, would need to be enhanced. For instance, a student may pay more attention and encode more information in memory when preparing for an exam that is crucial to a professional career. Here we investigate the neural mechanisms that underpin incentive motivation of mental versus physical efforts. More specifically, we ask whether mental and physical efforts are driven by a common, generic motivational center or if they are driven by distinct, dedicated modules.

The relationship between BOLD signal and task demand has appeared surprisingly simple. A repeated finding with functional MRI is that activity in task-specific regions increases with task difficulty. Indeed, more attention, cognitive control, or working memory load were related to greater hemodynamic signal in different regions of a prefronto-parietal network [2]–[6], whereas harder sensory discrimination and higher grip force were linked to greater signal in sensory and motor cortices [7]–[11]. The usual interpretation is that participants exert more effort when confronted with higher demands, resulting in more task-specific activation. Several of the above studies have manipulated task payoff in addition to task difficulty. Following on the same principle, task-specific regions were also modulated by expected rewards, reflecting the fact that participants exert more effort when there is more reward at stake. However, the functional link between task-specific and valuation regions remains poorly understood. The so-called brain valuation system (BVS), which was shown to express preferences, pleasantness ratings, and reward expectations [12]–[17], mainly includes the ventral prefrontal cortex and its basal ganglia (BG) target, the ventral striatum. These regions are also referred to as the limbic fronto-striatal circuit [18]–[21], in opposition to the cognitive circuit (including the dorsal prefrontal cortex and anterior caudate) and motor circuit (including the sensorimotor cortex and posterior putamen). The question is therefore whether motivating cognitive and motor effort involves the same or distinct areas of the limbic circuit.

**Figure 1:**
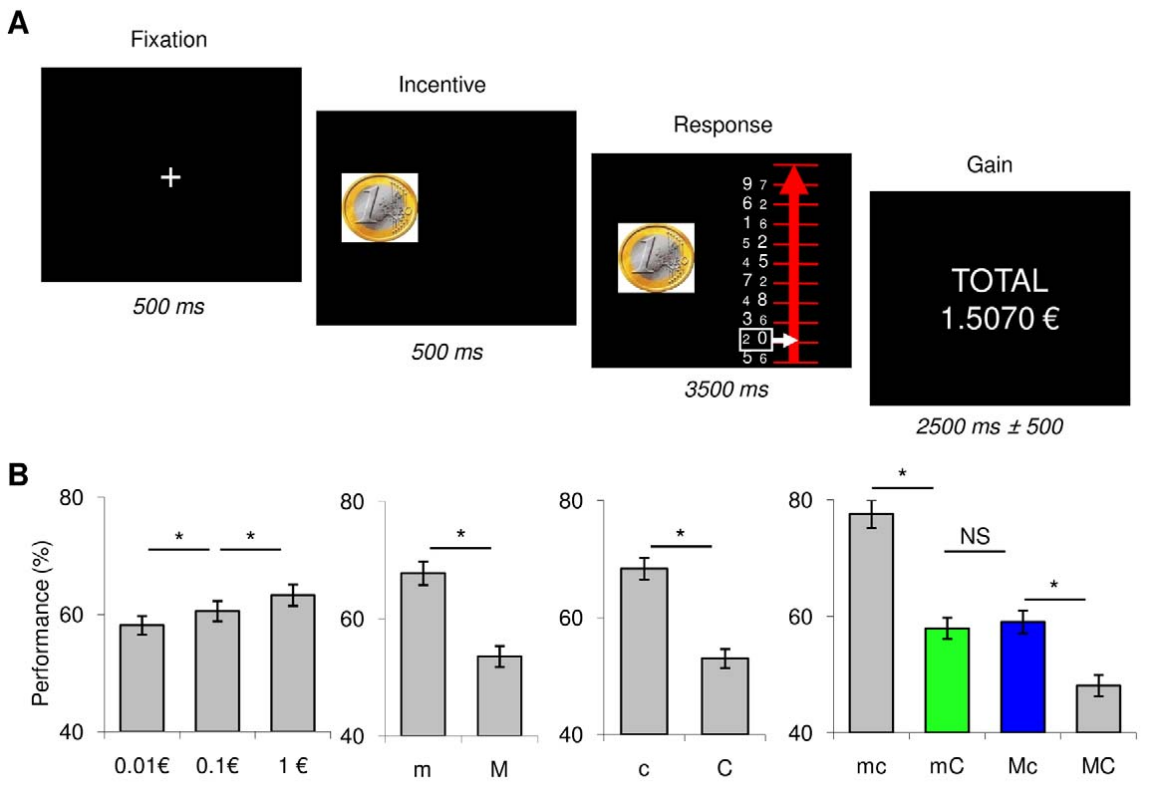
Behavioral task and results. (A) Example of task trial. Successive screenshots displayed are shown from left to right with durations in ms. Every trial started with a central fixation cross. Then the monetary incentive (0.01, 0.1, or 1€) was displayed as a coin image and effort was triggered by the onset of a graduated line representing a ladder. The goal was to move the white cursor up as high as possible, each step representing 10% of the money at stake. To reach the next step participants had to squeeze the handgrip on the side of the numerically greater figure in the white box. In congruent pairs this figure was also the greater physically (font size), whereas it was physically smaller in incongruent pairs. The motor demand was manipulated by changing the amount of force needed to reach the next step (30% versus 60% of the maximal force in easy versus hard trials). The cognitive demand was manipulated by changing the proportion of congruent pairs (100% versus 50% in easy versus hard trials). At the end of every trial the cumulative total of monetary earnings was displayed on the screen. (B) Performance across experimental conditions. Performance is expressed as the percentage of the monetary incentive reached (i.e., of steps completed on the ladder). Bars represent the average performance ± inter-participant standard error for the three monetary incentives (0.01, 0.1, and 1€) and the four effort conditions (m, easy motor effort; M, hard motor effort; c, easy cognitive effort; C, hard cognitive effort). * Significant difference (two-tailed paired *t* test, *p*<0.05); *ns*, non-significant.

In previous studies [10],[22] we identified the limbic BG (mostly the ventral striatum and pallidum) as responsible for motivating force production, which was underpinned by the motor cortex. The task consisted of squeezing a handgrip to win as much of various monetary incentives as possible. In the present functional MRI study, we combined this motor effort with a cognitive effort, related to interference monitoring in a numerical Stroop task. This task involves detecting the numerically greater figure within pairs. Incongruent pairs, where the numerically greater number is physically smaller, generate interferences that can only be overcome with sustained attentional effort. Functional MRI studies using numerical Stroop task showed that incongruent pairs yield more activations in specific prefrontal and parietal cortex areas [23]–[25]. Here, numerical comparisons indicated which handgrip (left or right) had to be squeezed in order to win 10% of the monetary incentive. Thus, participants had first to perform a numerical comparison (cognitive effort) and then to squeeze a handgrip (motor effort) to reach each of the 10 steps that lead up to the full amount of money at stake in a given trial (Figure 1A). On a trial-by-trial basis, motivation was varied by using different incentive levels (1c, 10c, 1€), cognitive effort by varying the proportion of congruent pairs (100% or 50%), and motor effort by varying the force to be produced (30% or 60% of maximal force). Note that 100% of congruent pairs and 30% of maximal force reduce cognitive and motor effort (respectively) to a minimum. Thus, within similar stimulus-response settings, the task demand could be either primarily motor or primarily cognitive.

To address our question, we investigated activity in brain regions reflecting incentive levels, cognitive, and motor demands. We reasoned that, if a common motivational node were to drive the two task-specific systems, its activity should (1) reflect incentive level whether the demand was motor or cognitive, (2) be correlated with the level of performance attained, and (3) switch connectivity between motor and cognitive circuits depending on the task demand.

## Results

### Behavioral Data

Global ANOVA showed a significant effect of the three main factors on task performance (proportion of steps completed on the ladder): monetary incentive (*F*_2,18_ = 4.06, *p*<0.05), cognitive demand (*F*_1,18_ = 88.04, *p*<0.001), and motor demand (*F*_1,18_ = 119.67, *p*<0.001). There was no significant interaction between incentive and difficulty levels and no triple interaction (*p*>0.9), indicating that motivation had a similar impact whether the limiting factor on performance was motor or cognitive. There was, however, an interaction between motor and cognitive difficulties (*F*_1,18_ = 14.80, *p*<0.001), indicating that their effects on behavioral performance were not additive. Post hoc comparisons showed that participants performed better when monetary incentives were larger (0.1 versus 0.01€: *t*_18_ = 3.48, *p*<0.01; 1 versus 0.1€: *t*_18_ = 4.09, *p*<0.001) and when the task was easier in terms of motor demand (m versus M: *t*_18_ = 6.97, *p*<0.001) and cognitive demand (c versus C: *t*_18_ = 13.24, *p*<0.001). Increase in performance was similar when removing the motor and cognitive difficulties (67.7%±2.0% versus 53.5%±1.8% and 68.3%±1.8% versus 53.0%±1.6%). As expected, the best performance was obtained when both motor and cognitive efforts were easy (mc: 77.6%±2.4%; mc versus mC: *t*_18_ = 13.04, *p*<0.001; mc versus Mc: *t*_18_ = 7.55, *p*<0.001) and the worst when they were both hard (MC: 48.1±1.8, MC versus Mc: *t*_18_ = 10.35, *p*<0.001; MC versus mC: *t*_18_ = 5.71, *p*<0.001). Importantly, performance for the two intermediate conditions (mC versus Mc) was similar (57.9%±1.8% versus 59.0%±1.9%). Thus, behavioral results show that the manipulation was successful at balancing effects of motor and cognitive demand (Figure 1B).

### Neuroimaging Data

All the activations apparent in statistical parametric maps (SPM) and described in the text survived family-wise error (FWE) correction over the entire brain, either at the voxel or cluster level (as indicated in figures).

**Figure 2:**
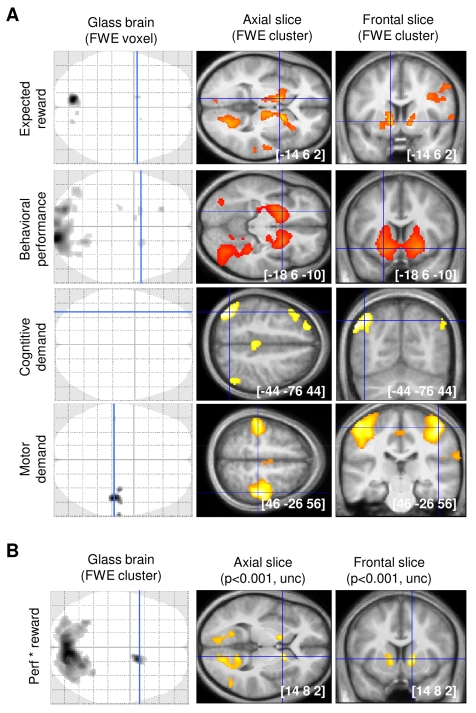
Neural correlates of main experimental factors and effects (reward level, cognitive and motor demands, and performance level). (A) Statistical parametric maps (SPM) show the main parametric modulation effects obtained with GLM1. (B) SPM shows the conjunction between expected reward and performance level modulation effects. Voxels displayed in grey-black on the glass brains survived a threshold of *p*<0.05 after voxel-wise correction for multiple comparisons (family-wise error, FWE). Frontal slices were taken at the maxima of interest indicated by blue lines on glass brains and superimposed on the average structural scan. Voxels displayed in yellow on slices survived a threshold of *p*<0.001 (uncorrected) after cluster-wise FWE correction for multiple comparisons. The [x y z] coordinates of maxima refer to the Montreal Neurological Institute (MNI) space.

In order to isolate the neural substrates that underpin incentive motivation, cognitive effort, and motor effort (Figure 2A), we designed a first general linear model (GLM1) that included separate regressors modeling the successive three events: incentive display with a delta function, effort exertion with a boxcar function, and outcome display with a delta function. These three categorical regressors were, respectively, modulated by the following parameters: expected reward (log-transformed incentive times average performance) for incentive display, performance level (height reached on the ladder), motor and cognitive demand (1 for high, 0 for low demand) for effort exertion, and monetary earning (log-transformed incentive times current performance) for outcome display. We first checked that motor and cognitive efforts recruited distinct neural networks. As expected, the motor demand was reflected in the primary sensorimotor cortex bilaterally, consistent with the fact that both hands were equally involved in all trials. Also expected, the cognitive demand was reflected in bilateral inferior parietal modules and left dorsolateral prefrontal cortex (DLPFC), with the addition of midline regions (paracingulate cortex).

Then we looked for the putative common motivational system, which must reflect expected reward and predict performance level. At incentive display, the expected reward was represented over distributed brain areas with a bilateral peak overlapping the ventral striatum (VS), internal capsule, and ventral pallidum. Other activation foci were located in the thalamus, insula, middle temporal, and posterior cingulate cortex. During the effort period, VS activity was also significantly correlated to the variability in behavioral performance. The conjunction between reward and performance effects (Figure 2B) yielded significant and more specific activation in the ventral striato-pallidal complex. The only other regions activated in relation to both expected rewards and performance levels were visual areas around the precuneus and calcarine sulci, which probably reflected the progression of the cursor on the computer screen. These results show that the VS reflects both the expected reward prior to effort exertion and the variability in performance that is not due to changes in task difficulty. To verify this pattern, we extracted the signal in 8-mm spheres centered on the left and right VS activation peaks obtained with the conjunction between expected reward and performance level. Post hoc analyses showed that VS activity (averaged between left and right clusters) significantly encoded reward and performance levels (both *p*<0.001) but not motor or cognitive task demand (both *p*>0.05). Finally, we did not find any activation related to monetary earnings at outcome display, even at a liberal threshold (*p*<0.001, uncorrected), possibly because they were fully predictable at that time.

**Figure 3:**
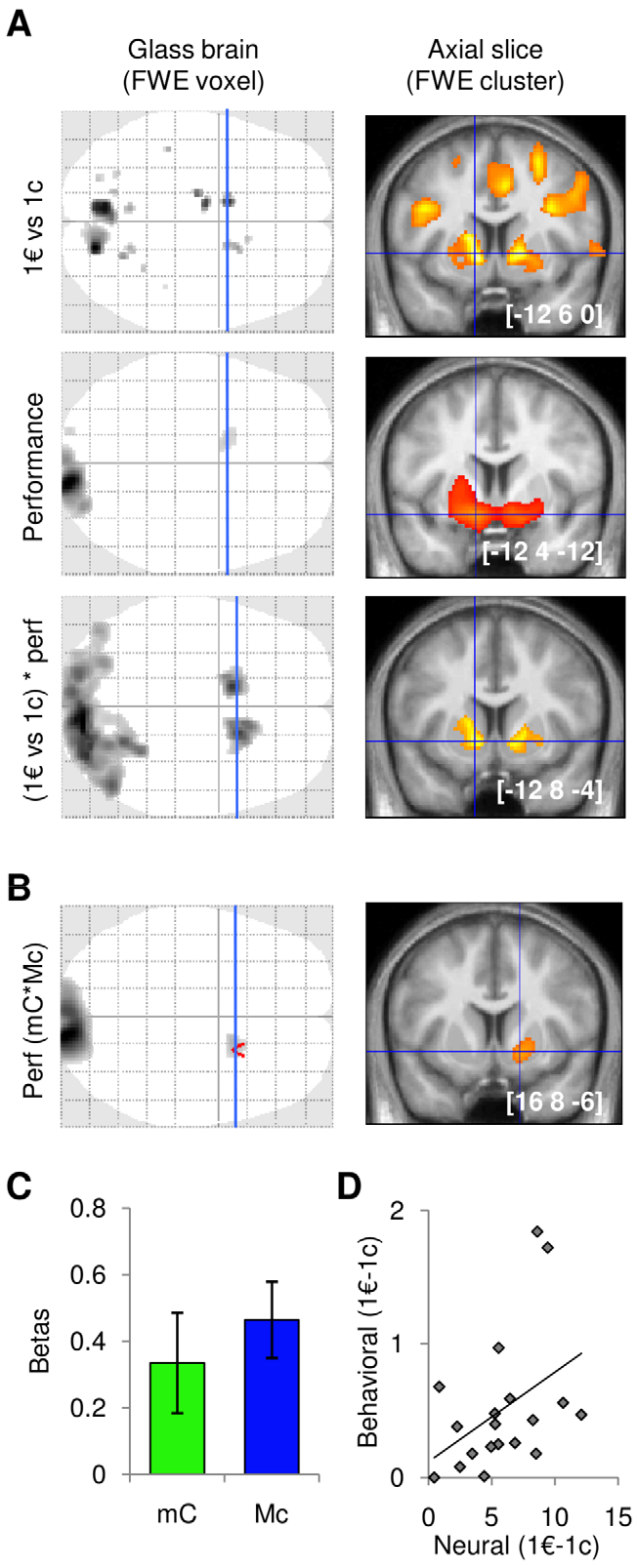
Neural correlates of performance effects depending on effort type (motor or cognitive demand). (A) SPM show activations obtained with GLM2 for the contrast between high and low incentives, the correlation with performance levels, and the conjunction between these two effects. (B) SPM shows regions parametrically modulated by performance level during both motor and cognitive effort (conjunction of mC and Mc conditions). (C) Graphs show regression coefficients (betas) obtained in the ventral striatum (VS) for the parametric modulation by performance levels during effort exertion in mC and Mc conditions, separately. Bars represent mean ± inter-participant standard errors. Dotted line indicates non-significant (NS) difference (two-tailed paired *t* test, *p*>0.05). (D) Scatter plots illustrate the inter-participant correlation between behavioral and neural incentive effects (1€ versus 1c). Solid line represents significant correlation (*p*<0.05).

That expected rewards are equally represented in the VS whatever the type of effort required (motor or cognitive) is rather trivial here, because participants had no information about the upcoming task at the time of incentive display. What is less trivial is that the effort type may not affect how VS activity reflects performance levels during task completion. To test this prediction we designed a second GLM (GLM2), in which the different conditions were modeled in separate regressors: three delta functions for incentive display (1c, 10c, and 1€), four boxcar functions for effort exertion (mc, mC, Mc, and MC), and one delta function for outcome display. The four regressors modeling effort exertion periods were parametrically modulated by performance levels. The VS was again significantly activated in the conjunction between incentive effect (1€ – 1c) and performance level, when collapsing all effort conditions (Figure 3A). We then focused on mC and Mc conditions, which present the same difficulty (and hence performance) but different types of effort (cognitive and motor, respectively). Conjunction analysis showed that the VS significantly reflected performance levels in both mC and Mc trials (Figure 2B). To test the alternative hypothesis that cognitive and motor efforts are motivated by different brain regions, we contrasted the effects of performance between mC and Mc conditions. These contrasts yielded no significant results, even at a more lenient threshold (*p*<0.001, uncorrected). Thus, we found a motivational system (with the VS as a main component) that is common to cognitive and motor efforts, but no motivational system that would be specific to one of them. We extracted the signal within the bilateral ROI defined by the conjunction between reward and performance effects to confirm that the performance attained was similarly represented in the VS whatever the type of effort required (i.e., for mC and Mc conditions, see Figure 3C).

So far we have shown that VS activity measured during effort exertion reflects (not predicts) performance levels. However, VS activity should not only reflects performance levels but also precede behavioral outputs, in order to be considered as causally responsible for motivating the behavior. We thus intended to provide evidence that VS activation measured before task completion predicts behavioral performance, using inter-participant variability. The contrast between high and low incentive display (1€–1c at the neuronal level) was extracted in the same VS ROI as above and correlated across participants with the effect of incentives on task performance (1€–1c at the behavioral level). Robust regression of neuronal against behavioral incentive effects was significant (beta = 2.69, *t*_18_ = 1.83, *p*<0.05), showing that VS sensitivity to increasing incentives predicts how much an individual will gain in performance (Figure 3C).

**Figure 4:**
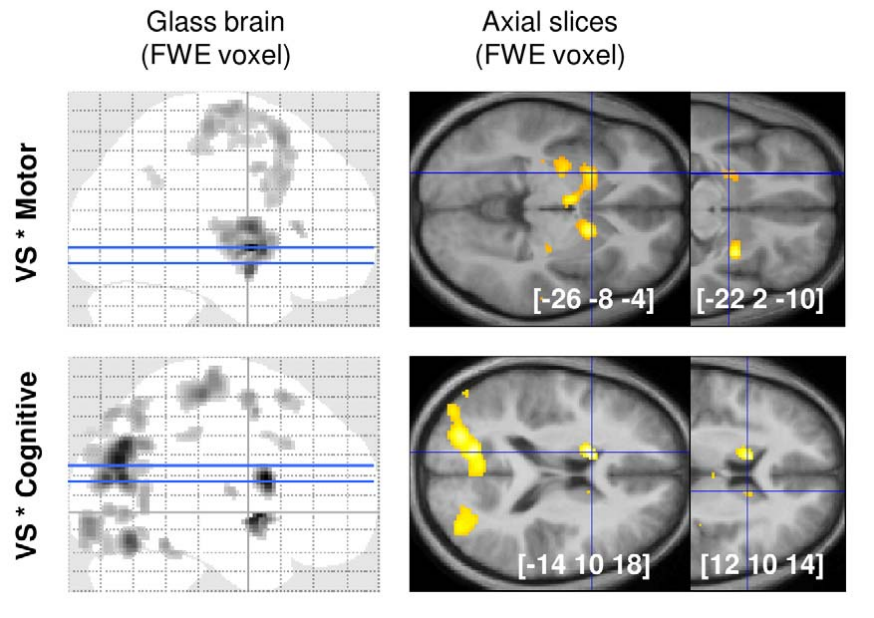
Psychophysiological interaction (PPI) between ventral striatum activity and task demand. SPMs show voxels in which activity was significantly correlated with left VS signal when the motor demand was high (top) and when the cognitive demand was high (bottom). Significant voxels (*p*<0.05, whole-brain voxel-wise FWE correction) are displayed in grey-black on glass brains and in orange-yellow on axial slices. Slices were taken at the peaks located in the left caudate for cognitive effort and in the left putamen for motor effort. Activations are superimposed on the average structural brain scan. The [x y z] coordinates refer to the Montreal Neurological Institute (MNI) space.

Last we investigated how this common motivational node could drive the activity in the two task-specific systems. We first ran a functional connectivity analysis to search for regions that were preferentially connected to the VS when the primary demand was cognitive versus motor. Specifically, we took the left VS as a seed and tested for psychophysiological interactions (PPI) with difficulty levels. We found that the VS was significantly connected with the caudate nucleus when the cognitive demand was high and with the putamen when the motor demand was high (Figure 4). Other regions included the thalamus, occipital, and parietal regions for motivation of cognitive effort, and the thalamus and bilateral precentral regions for motivation of motor effort. The caudate activation was more medial, anterior, and dorsal than the putamen activation, consistent with the distinction between cognitive and motor fronto-striatal circuits [18]–[21]. To complete this argument based on anatomical connectivity, we conducted another PPI analysis to demonstrate functional connectivity. We took 8-mm spheres positioned over caudate and putamen maxima (from the PPI results above) as simultaneous seeds and tested the interaction between their respective activity and the regressor modeling the effort period. The results were examined within 8-mm spheres centered on M1 and DLPFC maxima obtained in the regression with motor and cognitive demand, respectively (see illustration in Figure 2A). This ROI analysis showed that the putamen was more connected to M1 than to DLPFC (*t*_18_ = 2.28, *p*<0.05), and vice versa for the caudate nucleus (*t*_18_ = −2.51, *p*<0.05). Thus, our data suggest that the VS could switch functional connectivity between cognitive and motor regions depending on task demand.

**Figure 5:**
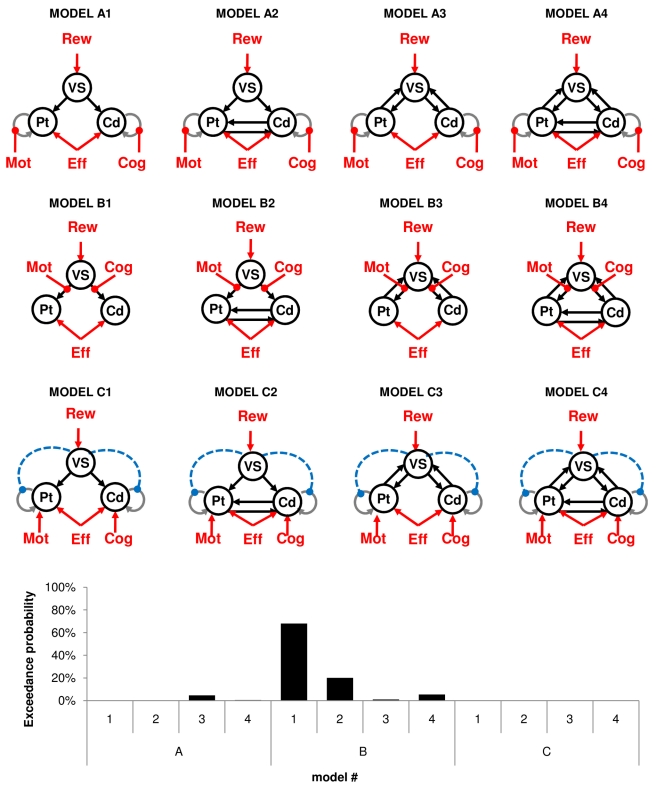
Dynamic causal modeling (DCM) analysis optimizing the connectivity structure of the striatal network. In all illustrated models, the driving inputs were boxcar functions over effort exertion periods (Eff) for the caudate (Cd) and putamen (Pt) and over incentive display plus effort exertion modulated by expected reward (Rew) for the ventral striatum (VS). From left to right, connections were systematically added up to a fully connected network. From top to bottom: the locus of parametric modulation by cognitive (Cog) and motor (Mot) task demand was varied. Graphs illustrate the result of a Bayesian model selection (BMS) procedure used to find the most likely model.

**Figure 6:**
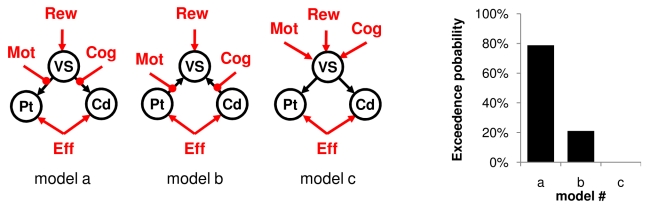
Dynamic causal modeling (DCM) analysis confronting our best model to alternative hypotheses. Model a is the winner of the previous Bayesian model selection (i.e., model B1 in Figure 5). Model b reversed the direction of links impacted by task demand modulatory, now going from caudate and putamen to the VS. Model c changed the loci of task demand modulatory effects, now affecting directly VS activity instead of connectivity. VS, ventral striatum; Cd, caudate nucleus; Pt, putamen; Rew, expected reward; Eff, effort exertion; Cog, cognitive demand; Mot, motor demand. Graphs illustrate the result of a Bayesian model selection (BMS) procedure, showing the exceedance probability of each model.

To further specify the directionality of interactions between neural activity and experimental factors within the basal ganglia, we finally conducted a dynamic causal modeling (DCM) analysis on our three striatal ROI (VS, caudate, and putamen). We first compared a series of models that were compatible with our GLM and PPI results. All these models contained at least two connections, from the VS to caudate and putamen. The driving inputs were boxcar functions covering both incentive display and effort exertion for the VS and effort exertion only for the caudate and putamen. VS activity was in addition parametrically modulated by the expected reward. The crucial difference between models resides in how the other experimental factors (cognitive and motor demand) influence the network. We tested three canonical interpretations of the PPI results (lines A to C, Figure 5). The first possibility (line A) is that cognitive and motor demands affect caudate and putamen self-connections, thereby modulating the sensitivity of these regions to VS input. The second possibility (line B) is that cognitive and motor demands directly modulate the strength of connections between VS and caudate or putamen. The third possibility (line C) is that motor and cognitive demands modulate caudate and putamen activity, to an extent that is modulated by the influence of VS activity on the self-connections of these regions. For each possibility we also varied the density of connections (columns 1 to 4, Figure 5), adding backward links from the caudate and putamen to VS, and/or bidirectional links between caudate and putamen. The most likely model identified using Bayesian model selection (BMS) was the simplest model implementing a direct modulation of forward links from VS to caudate and putamen by the cognitive and motor demands, respectively (model B1, Figure 5). Thus, the VS could mediate incentive effect on the relevant task-specific module, depending on task demand. In a second model comparison we confronted this winning model to other possible models in order to rule out alternative interpretations (Figure 6, top). One possible alternative is to reverse directionality, meaning that putamen and caudate activity would modulate the response of VS to expected rewards, depending on task demand. This may happen if VS response was an outcome (not a predictor) of behavioral performance. Another possible alternative is that task demand directly modulates VS activity, which would then integrate both costs and benefits. The BMS procedure assessed these two alternative models as less likely (Figure 6, bottom). Because in both BMS results the exceedance probability of the winning model was about 75%–80%, we cannot formally rule out the alternative models. These results nonetheless give credit to the idea that the VS could mediate incentive effects on both cognitive and motor efforts by boosting activity in either the caudate or putamen, depending on task demand.

## Discussion

We conducted a functional MRI study to examine whether mental and physical efforts rely on the same or distinct motivational systems in the human brain. We found that the same regions reflected the reward expected from both cognitive and motor effort exertion. Among these regions, the ventral striatum (VS) was the most prominent player. We further demonstrated two necessary characteristics for the VS to represent a common motivational node. First, VS activity predicted variations in behavioral performance that were not explained by task difficulty. Second, VS activity modulated cognitive regions during mental effort and motor regions during physical effort.

The three factors (incentive, cognitive, and motor levels) of our experimental design yielded activations in different brain systems, in accordance with the literature. Incentive levels were mostly reflected in the bilateral ventral striato-pallidum complex, which has recently emerged as a major reward-related region [26]–[30]. Incentive-related activations were not strictly specific to this region: they covered a large part of the brain, with secondary peaks in the bilateral thalamus, insula, middle temporal, and posterior cingulate cortex. Cognitive demand was mostly reflected in a prefronto-parietal network (dorsolateral prefrontal and inferior parietal cortex) that has been repeatedly implicated in cognitive control [23]–[25]. Motor demand was mostly reflected in the primary motor cortex, in keeping with previous studies on force production [7],[9],[11]. Thus, our experimental paradigm was successful in activating regions pertaining to the limbic, associative, and motor fronto-striatal circuits, in relation to incentive, cognitive, and motor aspects of the task. Apart from the cognitive effort, these results essentially replicate our previous findings that the ventral basal ganglia (striatum and pallidum mainly) reflect incentive level and the motor cortex the amount of force produced [10],[22].

However, that VS activity reflects expected reward is no proof that this region plays a motivational role. It could be that the VS is encoding reward expectation, irrespective of behavioral performance. To clarify this issue, we tested a regression with behavioral performance, which showed that VS activity was significantly linked to the step that participants reached within the ladder leading up to the full monetary incentive. Conjunction analysis showed that the same clusters in the ventral striatum and pallidum represented both expected reward and achieved performance. Correlations with reward and performance level were dissociable in time, since they were observed at incentive display and during effort exertion, respectively. To test whether the same regions were involved in motivating both types of effort we assessed the conjunction between conditions where the demand was primarily cognitive or motor. This conjunction identified clusters that significantly reflected performance level in both conditions, with a peak in the VS. To test whether any brain region would be involved in specifically motivating cognitive or motor effort, we examined contrasts between the two latter conditions. These contrasts yielded no significant activation, even with a liberal threshold that was uncorrected for multiple comparisons. Our data are therefore consistent with the hypothesis of a unique motivational system in the human brain that would have the ability to enhance both cognitive and motor efforts, via activation of task-dedicated regions.

It could also be argued that the VS reflects reward obtainment, which increased with both higher incentive level and better behavioral performance. However, we found no correlation between VS activity and monetary earnings at the time of feedback display. Note that monetary earnings could be predicted at that time, when knowing both incentive and performance level, which may explain the absence of neural response. We tested inter-participant correlations to show that VS activity could predict performance level before it is known, i.e. at the time of incentive display. We found that incentive effects on VS activity could predict subsequent incentive effects on behavioral performance. This is compatible with the idea that higher VS activity is the cause, not the consequence, of better behavioral performance. Another argument is that the amount of reward obtained varied as well with task difficulty, which was not encoded in VS activity. This result speaks against the VS encoding outcomes rather than expectations. It might suggest that the VS activation observed here is triggered by dopamine release, which was also shown to encode expected rewards but not action costs [31],[32].

The absence of effort representation in the VS is also interesting with respect to economic theories suggesting that benefits should be discounted by costs, and hence that motivation should decrease with task difficulty [33]. However, economic theories using discounted values aim at explaining choice, not effort exertion. Thus, the absence of cost representation may be due to the absence of choice in our paradigm: participants had to perform the task and did their best. In non-choice paradigms employed so far to demonstrate effort discounting in the VS [34],[35], monetary payoff was not dependent upon behavioral performance, which precludes investigating motivational processes occurring during effort exertion. Unlike economic theories, sport psychology may assume that task difficulty increases (not decreases) motivation [36]. Our data suggest that such performance motivation was not reflected in VS activity, which specifically mediated incentive motivation, i.e. the motivation arising from expected reward, with no influence of task demand. Note that participants were not explicitly informed about the cost, which they could only infer when performing the task. The absence of cost representation in the VS could therefore come from participants being unaware of task difficulty. Indeed, participants noticed that task demand varied with trials but could not explicitly report at debriefing that there were two difficulty levels for cognitive and motor efforts.

The next question was how the VS can boost behavioral performance. We addressed this question by investigating the psychophysiological interactions (PPI) between VS activity and task demand. We found that the VS was significantly connected with the cognitive regions (mostly the caudate) when cognitive demand was high, and with motor regions (mostly the putamen) when motor demand was high. This is consistent with the hypothesis that the VS represents a generic motivational node, driving cognitive circuits during mental effort and motor circuits during physical effort. We then assessed this hypothesis using dynamic causal modeling (DCM) analysis, focusing on interactions between striatal ROI (VS, caudate, and putamen) activity and experimental factors (incentive, cognitive, and motor demand). Bayesian model selection (BMS) procedures identified as most likely a model in which VS activity was modulated by expected rewards and in turn was driving caudate or putamen activity, depending on task demand. Importantly, the alternative DCMs included the possibilities that information on task demand directly affects the VS or is transmitted to the VS from the caudate and putamen. Because these three alternatives were assessed by BMS as less probable, we conclude that the most likely interpretation of our data is that the VS mediates the effects of incentives on the activation of relevant task-specific regions.

Several possibilities can be envisaged regarding the anatomical pathways linking the VS to cognitive and motor circuits. The PPI results highlighted striatal components and therefore suggest that the interaction occurs through basal ganglia (BG) intrinsic connections (possibly including with the thalamus). Notably, it is known that, at least at the pallidal level, dendrites are long enough to connect distant neurons that belong to different functional territories [37]. There are also pathways passing through midbrain dopaminergic populations that can connect the VS to more dorsal and posterior parts of the striatum [20]. It is less likely that the interaction occurs at the cortical level, as the ventral prefrontal cortex, which is the main cortical input to the VS, was not activated by monetary incentives in our results. This might relate to the simplicity of the valuation process: the same three stimuli were repeated throughout the task and they conveyed an over-learned meaning. Studies reporting activations of the ventral prefrontal cortex usually involve harder valuations, through associative learning, subjective feeling, or cost/benefit calculation [38]–[42]. Thus, our data are reminiscent of views considering the VS as a functional interface between motivation and action [43], driving the other BG territories when more reward is at stake.

We acknowledge that several questions remain to be clarified. A first issue is the spatial resolution of functional MRI. It remains possible that at a lower scale, different neuronal populations participate in different motivational processes. Voxel size was 2 mm in each plane, but surely we could not differentiate activation foci within the mid-height width of the Gaussian kernel used for spatial smoothing (8 mm). Single-unit recording in monkeys might help to further distinguish functional clusters, although this technique has not been very successful in circumscribing the topography of reward-related activities in the striatum. Previous single-unit studies reported that, even if more frequent in the VS, reward sensitivity could be observed in various parts of striatum, often in interaction with encoding of other task parameters [44]–[48]. It could be argued that the hemodynamic responses estimated with functional MRI give a better summary of the functional domain attached to a particular region. It is remarkable that the topography of hemodynamic responses matched the functional territories (limbic, cognitive, and motor) delineated using anatomical techniques such as axon tracing in monkeys or fiber tracking in humans [18],[20],[21],[49]. However, pharmacological micro-injection and high-frequency stimulation, in both human and non-human primates, have shown that close sites within basal ganglia nuclei can elicit markedly different behavioral effects [50]–[53]. One could speculate that more anterior or medial sub-regions of the VS would motivate cognitive processes, whereas more posterior or lateral sub-regions would motivate motor processes. We would still conclude that, at a macroscopic level, the VS is involved in motivating both cognitive and motor efforts.

A second issue is that correlation does not prove causality. DCM analyses enable assessing the probability of directional links [54], but proving that the VS was causally responsible for translating incentive into performance levels would require observing behavioral effects of VS inactivation. Interestingly, pharmacological inactivation of ventral striatal sites, using bicuculline microinjections, reduced spontaneous behaviors in monkeys [51]. In humans, bilateral basal ganglia damage, following vascular or anoxic strokes, can induce a so-called auto-activation deficit [55]. This syndrome is characterized by a dramatic reduction of spontaneous behavior, contrasting with a normal behavioral response to external instructions. In a previous study we showed that these patients are able to modulate their force according to external instructions but not depending on monetary incentives [56]. More specifically, valuation processes were preserved in these patients, as the skin conductance response reflected incentive level, but were not translated into physical effort. The lesioned areas could therefore be considered as causally responsible for translating higher expected reward into more physical effort—that is, for incentive motivation. However, lesions were not restricted to the VS, and they could affect various striatal and pallidal regions. One explanation is that any lesion interrupting connections between the ventral and dorsal parts of the striato-pallidal complex would prevent rewards to energize behavior. Thus, together with the present results, auto-activation deficit would make a case for a causal role of the VS in boosting the other BG circuits that underpin cognitive and motor functions.

In conclusion, we have developed a functional imaging paradigm capable of selectively activating components of the limbic, cognitive, and motor fronto-striatal circuits in relation to incentive motivation, mental, and physical effort. We found evidence that motivating mental and physical effort involves the VS driving the cognitive and motor circuits through local interactions. This conclusion calls for animal studies using electrophysiology to check these interactions at a lower scale and inactivation techniques to verify causality. One may also wonder whether this conclusion is compatible with studies investigating choice situations in which several tasks are available. Recent reports have shown a distributed representation of effector-specific option values [38],[57],[58], suggesting that each task value would be encoded in a distinct prefrontal region. To resolve the contradiction we may propose the following scenario: at the time of choice, task values would be represented in different prefrontal areas, but once the task is engaged its value is only represented in the striatum so as to drive task-specific regions. Obviously, demonstrating this speculative scenario would require further experimental work.

## Materials and Methods

### Participants

The study was approved by the Pitié-Salpêtrière Hospital ethics committee. Participants were recruited via email and gave informed consent prior to participating. A total of 20 participants (aged 19–27 years, 10 males/10 females, all right-handed) were scanned. Participants were screened for the following exclusion criteria: under 18 or above 39 years of age, currently taking drugs or medications, history of psychiatric or neurological illness, left-handedness, and contra-indications to MRI scanning (pregnancy, claustrophobia, metallic implants). One male participant with an important dolichocephaly was later excluded from data analysis, due to poor normalization to anatomical brain template. Participants believed that they would be playing for real money, but to avoid discrimination, payoff was rounded up to a fixed amount of 100€ for every participant.

### Behavioral Procedures

#### Experimental settings

Prior to scanning, participants were given written instructions to the task, which were repeated step by step orally. Subsequently, they were escorted inside the scanner and invited to find an optimal body position, while lying down with one power grip in each hand, the arms along the body. The power grips were made up of two molded plastic cylinders that compressed an air tube when squeezed (provided by the Wellcome Trust Centre for Neuroimaging, London, UK). The tube led to the control room, where it was connected to a transducer able to convert air pressure into voltage. Thus, compression of the two cylinders by an isometric handgrip resulted in the generation of a differential voltage signal, linearly proportional to the force exerted. The signal was fed to the stimuli presentation PC via a signal conditioner (CED 1401, Cambridge electronic design, UK). Stimuli presentation was programmed with Cogent 2000 (Wellcome Department of Imaging Neuroscience, London, UK). The visual stimuli were displayed behind the scanner on a projector screen, which participants could see via mirrors positioned over their eyes. Participants performed six task sessions in total (one practice and five test sessions). The practice session was done to familiarize participants with stimulus presentation and handgrip manipulation. Structural scans were acquired while participants were performing this practice session. Before starting each task session we calibrated the baseline (“just do nothing”) and measured the maximal force (“squeeze the grip as hard as you can”) for both hands. During these pre-tests, the dynamic changes of the recorded signal were used to provide participants with a real-time feedback of the force produced on the hand grip, which appeared as a red fluid moving up and down within a thermometer displayed on the computer screen.

#### Behavioral task

At the beginning of every task trial, participants had to fixate a central cross displayed on a computer screen (see Figure 1A). After 500 ms, the amount of money at stake was displayed as a coin image of 0.01, 0.1, or 1€. Coin images were displayed for practical reasons: it is a straight and efficient way to inform participants about the money at stake. We do not imply that motivational effects were due to Pavlovian associations between these cues and rewards. On the contrary, we believe that similar effects would be obtained if monetary incentives were indicated differently, for instance with Arabic figures. After incentive display (500 ms later), a graduated line representing a ladder appeared on the right of the coin image. Each graduation corresponded to a fraction (10%) of the monetary incentive and was associated with a pair of figures. The figures varied in both numerical size (between 1 and 9) and physical size (between two possible fonts). Thus the difference in physical size was the same for all pairs. The numerical difference varied from 1 to 5 (with two pairs of each in all trials). In congruent pairs, the numerically greater figure was also greater physically, whereas in incongruent pairs it was smaller. Incongruent pairs are known to generate a Stroop effect [23],[25] and hence require more attentional effort to inhibit interference and maintain accurate performance. New figures were presented on each trial such that participants could not anticipate them.

The task involved moving a white cursor as high as possible on the ladder, in order to win as much money as possible. To climb up one step of the ladder, participants had to squeeze either the left or right handgrip (with their left or right hand). The side was indicated by the numerically greater figure: if it was on the left (versus right), participants had to squeeze the left (versus right) handgrip. The pair of figures to be considered was highlighted by a white box. If the correct grip was squeezed above a given threshold, the white cursor moved one step up, indicating that 10% of the monetary incentive was won. Once the handgrip was released, the white box (around the figures) also moved one step up, highlighting the figures to consider for the next step. If the incorrect handgrip (on the wrong side) was squeezed, the cursor was frozen (could not be moved) for the rest of the response period. Thus, participants had to squeeze the correct grip and to release it to have access to the next step in the ladder. At the end of the trial, a cumulative total was displayed for 2,500 ms to indicate the money won so far. Random time intervals (jitters of ±500 ms) were inserted into every trial in order to ensure better sampling of the hemodynamic response and to avoid the sleepiness that can result from monotonous pace.

We independently manipulated the cognitive and motor demands in the task. The cognitive demand depended on the proportion of congruent pairs: there were 100% in easy trials and 50% in hard trials. The motor demand depended on the amount of force that had to be produced in order to move one step up: it was 30% of the maximal force for easy trials, and 60% for hard trials. The two types of difficulty (cognitive and motor) were crossed to form four conditions, referred to as mc, mC, Mc, and MC (lower case meaning easy and upper case hard). The four conditions were also orthogonal to monetary incentives. We therefore had a 3×2×2 factorial design: three monetary incentives (0.01, 0.1, and 1€), two cognitive demand levels (c and C: 100% and 50% of congruent pairs), and two motor demand levels (m and M: 30% and 60% of maximal force). The 12 conditions were randomly distributed over the trial series of each session, for a total of five repetitions (60 trials) and a duration of about 7 min.

#### Data analysis

Task performance was assessed as the graduation reached in the ladder (the proportion of 10% steps completed) at the end of the 3,500 ms time window. We did not consider error rates because they remained low (6.5% on average) and insensitive to task demand (m versus M: 6.0%±1.2% versus 7.0%±1.2%, *p* = 0.25; c versus C: 6.7%±1.2% versus 6.2%±1.1%, *p* = 0.44), probably due to a flooring effect. A global analysis of variance (ANOVA) was run to assess the effects of the three within-participant factors (monetary incentive, cognitive demand, and motor demand). Post hoc comparisons between monetary incentives and effort conditions were assessed across participants using two-tailed paired *t* tests. Three statistical significance thresholds were considered: *p*<0.05, *p*<0.01, and *p*<0.001. All statistical tests were done using the Matlab Statistical Toolbox (Matlab R2007b, The MathWorks Inc., USA).

### Imaging Procedures

#### Data acquisition

T2*-weighted echo planar images (EPI) were acquired with blood oxygen dependent level (BOLD) contrast on a 3.0 Tesla magnetic resonance scanner (Siemens Trio). A tilted plane acquisition sequence was employed to optimize functional sensitivity in the orbitofrontal cortex [59],[60]. To cover the whole brain with a TR of 1.830 s, we used the following parameters: 32 slices, 2 mm slice thickness, and 2 mm inter-slice gap. T1-weighted structural images were also acquired, co-registered with the mean EPI, segmented and normalized to a standard T1 template, and averaged across all participants to allow group-level anatomical localization. EPI images were analyzed in an event-related manner, within a general linear model, using the statistical parametric mapping software SPM8 (Wellcome Department of Imaging Neuroscience, London, UK). The first five volumes of each session were discarded to allow for T1 equilibration effects. Pre-processing consisted of spatial realignment, normalization using the same transformation as structural images, and spatial smoothing using a Gaussian kernel with a full-width at half-maximum of 8 mm. To correct for motion artifacts, participant-specific realignment parameters were included as covariates of no interest in all the general linear models (GLM) employed to analyze functional activations.

#### Neural activation

A first GLM (GLM1) was built to identify the brain regions related to the three experimental factors (monetary incentive, cognitive demand, and motor demand) plus the main effect of interest (performance attained). For each session, the GLM contained three separate regressors modeling the three main events of a trial, with a delta function for incentive display, a boxcar function for effort exertion period, and another delta function for outcome display. The length of boxcars was fixed to 3,500 ms, corresponding to the duration of effort exertion. The categorical regressors were parametrically modulated by expected reward (log-transformed incentive times performance averaged over all previous trials) for incentive display, motor and cognitive demand (both coded as binary variables) plus performance level (proportion of steps climbed in the ladder) for effort exertion, and monetary earning (log-transformed incentive times current performance level) for outcome display.

A second GLM (GLM2) was built to examine how representation of performance varied with the type and difficulty of the task. The different experimental conditions were modeled as separate categorical regressors, which avoided the issue of how orthogalization order affects the estimation of parametric regressors. Thus, this second GLM included 13 regressors of interest: three for the three incentive levels modeled as delta functions aligned to incentive display, eight for the four effort types (mc, mC, Mc, and MC) modeled as boxcar functions over effort exertion periods, each modulated by performance level, and two for outcome display modeled as a delta function modulated by the money won.

For both GLM, all regressors of interest were convolved with the canonical hemodynamic response function (HRF), combined with its first time derivative. Contrasts of regression coefficients (betas) were set over the canonical HRF at the individual level. Linear contrasts were then taken to a group-level random-effect analysis, using one-sample *t* tests. All illustrated activations survived a threshold of *p*<0.05 after family-wise error (FWE) correction for multiple comparisons over the whole brain, at the voxel level for glass brains, and at the cluster level for slices (minimum of 160 voxels).

Regression coefficients (betas) were extracted at the individual level from regions of interest (ROI), which were defined as 8-mm spheres positioned over maxima of interest observed in group-level SPM obtained with the first GLM. For every participant the betas were averaged over all the voxels within the ROI. Individual betas were then used for post hoc comparisons between experimental conditions (using one-tailed paired *t* tests) and for correlation with experimental variables (using robust regression test).

#### Functional connectivity

The psychophysiological interaction (PPI) analysis was based on a third GLM close to GLM2, where incentive display was modeled by a single regressor modulated by expected reward and where parametric modulations by task performance were removed. Signal in the left VS was extracted from a 8-mm diameter sphere centered on the peak of VS clusters correlated with expected reward (MNI coordinates: [−10 4 −2]). Motor effort was modeled as a succession of 3,500-ms boxcars over effort exertion periods for trials involving high motor demand (combining MC and Mc regressors). Symmetrically, cognitive effort was modeled as a succession of 3,500-ms boxcars over effort exertion periods for trials involving high cognitive demand (combining MC and mC regressors). Following standard PPI procedure [61],[62], the VS signal was first deconvolved and then multiplied by the cognitive and motor effort regressors to obtain interaction terms. The five regressors (motor interaction, cognitive interaction, VS signal, motor boxcars, and cognitive boxcars) were then convolved with a canonical HRF and entered in a first-level GLM for each participant.

We also used the GLM built for this PPI analysis to assess functional connectivity between striatal and frontal regions pertaining to the motor and cognitive circuits. Signal was extracted from caudate and putamen ROI defined as 8-mm diameter spheres centered on maxima of interest observed in group-level PPI results (MNI coordinates: [−14 10 18] for the caudate nucleus and [−26 −8 −4] for the putamen). These signals were deconvolved and multiplied by a boxcar function signaling effort period (regardless of the type of effort required). These two regressors representing interaction terms were then convolved with a canonical HRF and entered in a first-level GLM for each participant. The rest of the procedure was identical to the previous PPI. Regression coefficients (betas) estimated for the interaction terms were extracted and compared within 8-mm diameter spheres centered on frontal activation related to cognitive and motor demand (MNI coordinates: [−50 −26 58] for DLPFC and [−50 −26 58] for M1).

#### Effective connectivity

Modulation of intra-striatal effective connectivity by experimental factors was assessed using dynamic causal modeling (DCM) analysis, as implemented in SPM8. The GLM built for this analysis contained the following regressors: a boxcar function encompassing both incentive display and effort exertion, modulated by the expected reward (Rew), a boxcar function over all effort periods (Eff), and two boxcar functions modeling effort periods where primary demand was cognitive (Cog) or motor (Mot). Regressor Rew was meant to represent both the driving input and parametric modulation accounting for the VS activation dynamics. Regressor Eff was a common driving input for task-dedicated regions (caudate and putamen). Mot and Cog were meant to model parametric modulation by task demand. All categorical and parametric regressors were convolved with a canonical HRF.

After GLM estimation using SPM8, the signal was extracted from 8-mm spheres centered on the group-level maxima obtained with parametric modulation by Rew for the VS and with the PPI interaction regressors for the caudate and putamen (as illustrated in Figure 4). Following established procedure [63], we first optimized the connectivity structure of our network. Starting with a minimal architecture including only two unidirectional links from the VS to caudate and putamen, we systematically added connections up to a full connectivity network (from left to right columns, Figure 5). For each network we included three variants that differ on the target of Cog and Mot regressors: putamen and caudate self-connections (line A), forward links from VS to caudate and putamen (line B), or directly caudate and putamen activity (line C). For the latter we added a modulation of caudate and putamen self-connections by VS activity, such that the three variants corresponded to canonical interpretations of the PPI results. The most likely model was identified using a standard Bayesian model selection (BMS) procedure [64].

Once the intrinsic endogenous connections had been optimized, the most likely model was tested against two alternative models, to further establish its specificity. The first alternative model reversed the directionality of connections impacted by Cog and Mot regressors, with VS activity being driven by either the caudate or putamen, depending on task demand. The second alternative model changed the locus of parametric modulations, with the VS integrating both expected reward and task demand before sending this integrated information to both the caudate and putamen. Again, a BMS procedure was used to compare these alternative models to previous ones.

## Abbreviations

BG: basal ganglia
BMS: Bayesian model selection
BOLD: blood oxygen dependant level
BVS: brain valuation system
DCM: dynamic causal modeling
DLPFC: dorsolateral prefrontal cortex
FWE: family-wise error
GLM: general linear model
HRF: hemodynamic response function
M1: primary motor cortex
MNI: Montreal Neurological Institute
MRI: magnetic resonance imaging
PPI: psychophysiological interactions
ROI: region of interest
SPM: statistical parametric map
VS: ventral striatum
